# Identifying determinants of under-five child mortality in northern Togo

**DOI:** 10.7189/jogh.14.04019

**Published:** 2024-02-02

**Authors:** Samantha R Levano, John Kraemer, Désiré Dabla, Essodinam Agnes Miziou, Jessica Haughton, Heidi E Jones, Chloe Teasdale, Didier Ekouevi, Lisa R Hirschhorn, Kevin P Fiori

**Affiliations:** 1 Department of Pediatrics, Albert Einstein College of Medicine, Bronx, New York, USA; 2 Community Health Systems Lab, Integrate Health/Santé Intégrée, Bronx, New York, USA/Kara, Togo; 3 Department of Family & Social Medicine, Albert Einstein College of Medicine, Bronx, New York, USA; 4 Department of Health Management and Policy, Georgetown University School of Health, Washington D.C., USA; 5 Department of Epidemiology and Biostatistics, City University of New York Graduate School of Public Health and Health Policy, New York, USA; 6 CUNY Institute of Implementation Science in Population Health, New York, USA; 7 Department of Public Health, Health Sciences Faculty, University of Lomé, Lomé, Togo; 8 African Research Center in Epidemiology and Public Health, Lomé, Togo; 9 Feinberg School of Medicine, Northwestern University, Chicago, Illinois, USA

## Abstract

**Background:**

Although global rates of under-five mortality have declined, many low- and middle-income countries (LMICs), including Togo, have not achieved sufficient progress. We aimed to identify the structural and intermediary determinants associated with under-five mortality in northern Togo.

**Methods:**

We collected population-representative cross-sectional household surveys adapted from the Demographic Household Survey (DHS) and Multiple Indicator Cluster Survey from women of reproductive age in northern Togo in 2018. The primary outcome was under-five mortality for children born to respondents in the 10-year period prior to the survey. We selected structural and intermediary determinants of health from the World Health Organization Conceptual Framework for Action on the Social Determinants of Health. We estimated associations between determinants and under-five mortality for births in the last 10 years (model 1 and 2) and two years (model 3) using Cox proportional hazards models.

**Results:**

Of the 20 121 live births in the last 10 years, 982 (4.80%) children died prior to five years of age. Prior death of a sibling (adjusted hazard ratio (aHR) = 5.02; 95% confidence interval (CI) = 4.23–5.97), maternal ethnicity (i.e. Konkomba, Temberma, Lamba, Losso, or Peul), multiple birth status (aHR = 2.27; 95% CI = 1.78–2.90), maternal age under 25 years (women <19 years: aHR = 2.05; 95% CI = 1.75–2.39; women 20–24 years: aHR = 1.48; 95% CI = 1.29–1.68), lower birth interval (aHR = 1.51; 95% CI = 1.31–1.74), and higher birth order (second or third born: aHR = 1.45; 95% CI = 1.32–1.60; third or later born: aHR = 2.14; 95% CI = 1.74–2.63) were associated with higher hazard of under-five mortality. Female children had lower hazards of under-five mortality (aHR = 0.80; 95% CI = 0.73–0.89). Under-five mortality was also lower for children born in the last two years (n = 4852) whose mothers received any (aHR = 0.48; 95% CI = 0.30–0.78) or high quality (aHR = 0.51; 95% CI = 0.29–0.88) prenatal care.

**Conclusion:**

Compared to previous DHS estimates, under-five mortality has decreased in Togo, but remains higher than other LMICs. Prior death of a sibling and several intermediary determinants were associated with a higher risk of mortality, while receipt of prenatal care reduced that risk. These findings have significant implications on reducing disparities related to mortality through strengthening maternal and child health care delivery.

Global rates of child mortality have significantly declined since 1990 with continued progress made towards the Sustainable Development Goal (SDG) target of less than 25 under-five deaths per 1000 live births by 2030 [1]. Several low- and middle-income countries (LMICs), however, are expected to fall short of this target due to disparities in managing preventable and treatable diseases [2–4]. According to the World Health Organization (WHO), leading causes of under-five mortality include pneumonia, diarrhoea, and malaria followed by pre-term birth complications, birth asphyxia, and congenital anomalies [5]. Access to key maternal and child health services, ranging from skilled care at delivery to early childhood vaccination, has the potential to address these health disparities and save millions of children.

Previous studies have demonstrated that child mortality is driven by complex interplays of socioeconomic and biological determinants [6]. In a recent analysis of Demographic Household Survey (DHS) data in sub-Saharan Africa, higher under-five mortality was associated with socioeconomic determinants such as place of residence, maternal and paternal education, and access to safe water, as well as biological determinants such as lower birth interval, higher birth order, lower birth weight, multiple birth status, and lower maternal age [7,8]. Place of delivery (i.e. home or health facility) was also identified as a predictor of infant and under-five mortality in the previously described analyses [7,8]. Interventions focused on prenatal care, facility-based skilled care at childbirth, emergency obstetric postnatal care, child immunisations, nutrition support, infectious diseases prevention, and primary care have been recommended to reduce maternal and child mortality [9]. Despite substantial improvement in coverage of essential maternal and child health services in sub-Saharan Africa in recent decades, differences in health care access and utilisation persist, which likely contribute to the lack of the progress towards SDG targets [9,10].

Togo, a country in West Africa, will likely fall short of the SDG targets, with recent reports of under-five mortality by the United Nations Inter-agency Group for Child Mortality Estimation (IGME) indicating a rate of 68.8 deaths per 1000 live births in 2018 [11]. Additional estimates from the 2013 DHS demonstrated worse progress towards reduced under-five mortality in the northern region of Kara, which reported 130 deaths per 1000 live births compared to the national rate of 88 deaths per 1000 live births at the time [12]. The DHS also identified malaria, actuate lower respiratory infections, and diarrheal diseases as primary causes of under-five mortality in Togo, and rural residence, lower maternal education, younger maternal age, lower wealth status, child male sex, higher birth order, birth interval less than two years, and small size at birth as determinants of mortality [12]. Additional determinants such as previous death of a sibling and place of delivery appear understudied across sub-Saharan Africa, with little to no research available in Togo [6,13].

Togo has a fragmented health care system, driven largely by a fee-for-service structure [12]. The Ministry of Health in Togo developed a national health plan to align global best practices with national policy; however, children continue to die from diseases with known effective and low-cost treatments [3,14]. There are only five doctors for every 100 000 people in Togo, 70% of whom practice in the capital city, Lomé [12]. Access to health care is limited, with only 30% of the population in Togo reporting using public health facilities, despite 62% living within five kilometers of these facilities [12].

Integrate Health, an international nongovernmental organisation, has collaborated closely with the Togolese Ministry of Health to accelerate progress toward improving maternal and child health and addressing gaps in care delivery in northern Togo since 2014. In 2018, Integrate Health developed an integrated primary care programme that aimed to increase uptake of evidence-based interventions to improve maternal and child health in four rural districts in the Kara region of northern Togo [15]. We aimed to identify structural and intermediary determinants associated with under-five mortality in northern Togo to inform key priority areas and accelerate progress towards SDG targets.

## METHODS

### Study design

We conducted a pragmatic type II hybrid effectiveness-implementation study to evaluate Integrate Health’s integrated primary care programme intervention. Population-representative cross-sectional household surveys were designed and administered between May and July 2018 as a pre-intervention baseline for this ongoing five-year study. Data included in this analysis were collected from female residents aged 15–49 years residing in the catchment areas of 21 public sector health centres. Data collectors aimed to survey 7600 of the over 181 000 residents in the study region [15]. Households were selected randomly using a systematic random sampling strategy without replacement. Data collectors selected households from within each catchment area using a pre-calculated sampling fraction (i.e. every 1/2 households selected) based on previous census estimates and satellite imagery. If more than one eligible female resided in a selected household, participants were then chosen using a Kish selection grid method [16].

We adapted the household survey from the 2013 DHS and 2010 Multiple Indicator Cluster Survey (MICS) implemented in Togo. Trained staff collected data using electronic tablets with questionnaires developed in KoBoToolbox [17]. Surveys were conducted in either French or the local language, depending on respondent preference. The local languages spoken in northern Togo are not widely taught as written languages; therefore, the surveys were only validated in French. For participants with limited French proficiency, data collectors sight-translated the validated survey and read the questions aloud in the relevant local language. Data collected included measures of demographic characteristics, childhood illness prevalence, health service coverage, barriers to care, and health-seeking behavior. The Institutional Review Boards of the Togolese Ministry of Health in Lomé, Togo (ref: CRBS/33/2017) and the Albert Einstein College of Medicine in New York, USA (ref: 2017-8411) approved the study. All respondents provided written informed consent.

### Study measures

The primary outcome of interest in this study was under-five mortality, defined as child death before the age of five years. Female participants reported their live births in the previous 10 years, including births for children alive and deceased at the time of interview. Data collected for each child included date of birth, sex, status at time of interview (e.g. alive or deceased), and age at death, if relevant.

We selected potential independent determinants of child mortality from the WHO Conceptual Framework for Action on the Social Determinants of Health, within which structural determinants, known as the social determinants of health, operate through a set of intermediary determinants to shape health outcomes [18]. The WHO identifies material circumstances (e.g. living and working conditions, food availability, etc.), biological factors, and psychosocial factors as the primary intermediary determinants, which are considered distinct from social determinants. Additionally, the health system itself is considered an intermediary determinant in the Conceptual Framework because of its pivotal role in increasing access to care and promoting intersectoral action to improve health outcomes.

**Figure 1:**
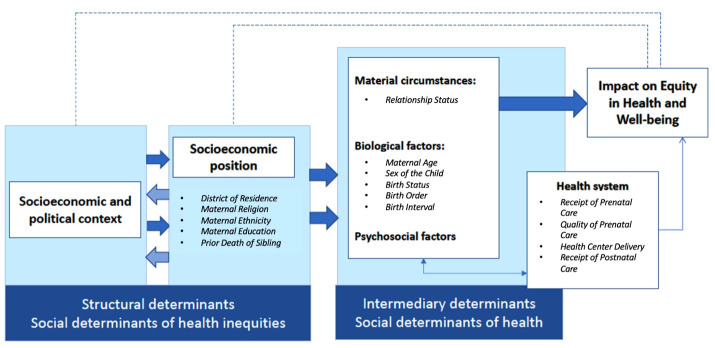
Conceptual framework adapted from the WHO Conceptual Framework for Action on the Social Determinants of Health.

We categorised structural socioeconomic determinants (e.g. district of residence, maternal religion, maternal ethnicity, maternal education, prior death of a sibling), intermediary material circumstances (e.g. relationship status), intermediary biological factors (e.g. maternal age, sex of the child, birth status, birth order, birth interval), and proxies for health system utilisation (e.g. prenatal care, quality of prenatal care, health centre delivery, postnatal care) based on previous literature and available measures in the household survey (**Figure 1**). Psychosocial factors were not available in the household survey and were not included in this analysis. Additional data collected for wealth quintile and health insurance status were measured at the time of the survey but may have changed over the 10-year period of reported births; therefore, we excluded these measures in our analysis of under-five mortality.

Proxies for health system measures represented utilisation by the mother during her pregnancy with the child (e.g. prenatal care, quality of prenatal care, health centre delivery) or utilisation by the child after birth (e.g. postnatal care for infants). High-quality prenatal care was defined as attending four or more visits with a skilled provider, being tested for HIV during pregnancy, taking medication to avoid contracting malaria during pregnancy, and receiving examinations for blood pressure, urine, and blood collection at least once during reported prenatal visits. Measures of health system utilisation were only collected for children born in the two years prior to the survey date, which are considered recent births.

### Statistical analysis

We calculated sampling weights to account for the probability of selection and non-response based on the number of households approached and number of eligible women per household within each cluster and site (i.e. 16 clusters each for 21 sites total). We calculated descriptive statistics, including raw counts and weighted population percentages, in Stata, version 2017 (StataCorp LLC, College Station, TX, USA) using the survey commands available through the ‘svy’ and ‘svyset’ packages with incorporation of the applicable population-based sampling weights [19]. We compared distribution of demographic characteristics were compared for female respondents with births in the last 10 years and those with births in the last two years, which reflect the inclusion criteria selected for the analysis of socioeconomic and biological determinants and health system utilisation, respectively.

We used Cox proportional hazards models to examine the association of our pre-selected determinants of interest. Follow up time for each child was estimated as: age in months for children who were alive and <5 years of age at the time of the survey; age in months was set to 60 months for children who were ≥5 years at the time of the survey; and age at death in months for children who died <5 years of age. We developed descriptive statistics, hazards models, and survival curves using the ‘svy,’ ‘stset,’ ‘stcox,’ and ‘stcurve’ packages in Stata, Stata, version 2017 (StataCorp LLC, College Station, TX, USA) while accounting for sampling weights [20]. Survival curves use the survivor function, which is based on the fitted Cox proportional hazards models reported.

We used unadjusted and adjusted analyses for each model to assess independent determinant associations with under-five mortality. Model 1 examined socioeconomic (e.g. district, maternal religion, maternal ethnicity, maternal education) and biological predictors (e.g. maternal age, sex, birth status, birth order) for all births reported in the last 10 years. Adjusted hazards ratios (aHRs) described the relative risk of with under-five mortality when controlling for all predictors of interest.

Additional data were collected for second or later order births (i.e. non-first born children) reported in the last 10 years, including measures for prior death of a sibling and birth interval. Model 2 excluded first born children (n = 9091) and assessed all socioeconomic (e.g. district, maternal religion, maternal ethnicity, maternal education, prior death of sibling under five) and biological predictors (e.g. maternal age, sex, birth status, birth order, birth interval) of interest.

Health system utilisation measures were only collected for births in the last two years. Model 3 excluded births reported more than two years prior to the survey (n = 15 269) and births reported less than two years prior to the survey with missing values (n = 264). The following utilisation measures were examined in Model 3: prenatal care during pregnancy of recent birth, quality of prenatal care during pregnancy of recent birth, health centre delivery for recent birth, and postnatal care for infant.

## RESULTS

### Study population

Of the 10 834 households with eligible women selected, 10 753 (99.2%) completed or partially completed the survey. There were 20 121 live births reported in the 10 years prior to the survey, with 4852 reported in the previous two years. Most respondents who reported live births in the last 10 years were over the age of 25 years, belonged to the traditional/animist religious group, and did not have formal schooling (**Table 1**).

**Table 1:** Demographic characteristics and intermediary material determinants of survey participants by recent birth status in northern Togo, 2018

Maternal characteristic by selected households, n (%)*	Respondents with birth in last 10 y (n = 8949)	Respondents with birth in last two y (n = 4761)
District		
*Binah*	1714 (21.78)	857 (20.76)
*Bassar*	2071 (21.28)	990 (19.46)
*Dankpen*	2443 (26.01)	1415 (27.56)
*Keran*	2721 (30.94)	1499 (32.21)
Maternal age group		
*15–17 y*	63 (0.81)	47 (1.19)
*18–24 y*	2118 (24.15)	1505 (32.34)
*25–49 y*	6768 (75.04)	3209 (66.47)
Maternal education level completed		
*None*	5481 (61.27)	2785 (57.84)
*Primary*	2392 (26.75)	1325 (28.35)
*Secondary or above*	1076 (11.98)	651 (13.81)
Relationship status		
*Single*	323 (3.67)	97 (2.24)
*Married or living together, without co-wives*	5555 (54.36)	3074 (56.68)
*Married or living together, with co-wives*	3071 (41.97)	1590 (41.08)
Maternal ethnicity		
*Kabiye*	1649 (19.25)	789 (17.72)
*Konkomba*	2594 (27.84)	1484 (29.18)
*Temberma*	1619 (18.10)	894 (18.40)
*Lamba*	1255 (14.29)	675 (15.06)
*Bassar*	459 (4.54)	196 (3.58)
*Losso*	513 (5.45)	246 (5.02)
*Peul*	348 (3.96)	211 (4.56)
*Sola*	250 (3.60)	118 (3.21)
*Other*	262 (2.96)	148 (3.28)
Maternal religion		
*Animist*	5817 (63.46)	3094 (63.35)
*Muslim*	654 (7.73)	375 (8.46)
*Christian*	2124 (24.82)	1105 (24.22)
*No religion*	354 (3.99)	187 (3.97)
Wealth index		
*Q1 (poorest)*	1833 (18.66)	919 (17.04)
*Q2 (poorer)*	1833 (19.27)	999 (19.60)
*Q3 (middle)*	1762 (20.06)	955 (20.62)
*Q4 (wealthier)*	1868 (22.10)	1005 (22.87)
*Q5 (wealthiest)*	1653 (19.89)	883 (19.87)
Health insurance		
*No*	8474 (95.00)	4563 (96.10)
*Yes*	475 (5.00)	198 (3.90)

q – quintile, y – years

*Presented as actual sample sizes with weighted percentages.

### Structural socioeconomic determinants associated with under-five mortality

Of the 20 121 live births in the last 10 years, 982 (4.80%) children died prior to five years of age. In model 1, maternal ethnicity was the only structural socioeconomic determinant associated with under-five mortality. Children born to women who identified as Konkomba, Temberma, Lamba, Losso, Peul, or Other had higher hazards of under-five mortality compared to children of Kabiye women (**Table 2**).

**Table 2:** Model 1, socioeconomic and biological determinants of under-five mortality for all births in northern Togo in the last 10 y (2008–18)

	Births in the last 10 y (n = 20 121 (100.00%)), n*	Deaths under-five y (n = 982 (4.78%)), n (%)*	Unadjusted hazard ratio (95% CI)	Adjusted hazard ratio (95% CI)†
**Structural/socioeconomic determinants**				
District				
*Binah*	3479	107 (3.31)	1.00 (ref)	1.00 (ref)
*Bassar*	4614	200 (4.68)	1.39 (1.10–1.75)	0.84 (0.55–1.30)
*Dankpen*	6139	409 (6.52)	1.97 (1.61–2.41)	0.93 (0.58–1.49)
*Keran*	5889	266 (4.09)	1.26 (1.01–1.56)	0.65 (0.42–1.02)
Maternal religion				
*Animist*	13 473	684 (4.96)	1.00 (ref)	1.00 (ref)
*Muslim*	1398	51 (4.16)	0.86 (0.63–1.17)	0.75 (0.50–1.11)
*Christian*	4451	214 (4.72)	0.96 (0.83–1.11)	1.04 (0.89–1.21)
*No religion*	799	33 (3.36)	0.67 (0.48–0.94)	0.66 (0.47–0.92)
Maternal ethnicity				
*Kabiye*	3396	93 (2.97)	1.00 (ref)	1.00 (ref)
*Konkomba*	6530	429 (6.50)	2.21 (1.82–2.69)	2.20 (1.42–3.41)
*Temberma*	3562	178 (4.43)	1.53 (1.23–1.90)	2.09 (1.36–3.20)
*Lamba*	2722	118 (4.18)	1.44 (1.12–1.85)	1.84 (1.21–2.82)
*Bassar*	939	33 (3.46)	1.13 (0.82–1.56)	1.40 (0.87–2.24)
*Losso*	1169	54 (5.32)	1.78 (1.28–2.48)	2.01 (1.25–3.23)
*Peul*	805	33 (5.10)	1.77 (1.15–2.72)	2.34 (1.16–4.73)
*Sola*	459	21 (3.15)	1.08 (0.66–1.76)	1.04 (0.64–1.71)
*Other*	539	23 (4.11)	1.44 (0.92–2.24)	1.81 (1.12–2.93)
Maternal education level completed				
*None*	13 023	674 (5.13)	1.00 (ref)	1.00 (ref)
*Primary*	5185	239 (4.32)	0.87 (0.76–0.99)	0.92 (0.79–1.06)
*Secondary or above*	1913	69 (3.66)	0.78 (0.62–0.99)	0.83 (0.63–1.08)
**Intermediary biological determinants**				
Maternal age at time of birth				
*≥25 y*	11 501	474 (4.08)	1.00 (ref)	1.00 (ref)
*20–24 y*	5751	313 (5.43)	1.31 (1.15–1.49)	1.48 (1.29–1.68)
*≤19 y*	2869	195 (6.36)	1.57 (1.36–1.82)	2.05 (1.75–2.39)
Sex of the child				
*Male*	10 355	556 (5.25)	1.00 (ref)	1.00 (ref)
*Female*	9766	426 (4.29)	0.82 (0.74–0.90)	0.80 (0.73–0.89)
Birth status				
*Singleton*	19 454	923 (4.62)	1.00 (ref)	1.00 (ref)
*Multiple birth*	667	59 (9.21)	2.12 (1.66–2.71)	2.27 (1.78–2.90)
Birth order				
*1*	9091	410 (4.36)	1.00 (ref)	1.00 (ref)
*2–3*	9652	495 (5.10)	1.32 (1.20–1.45)	1.45 (1.32–1.60)
*>3*	1378	77 (5.49)	1.96 (1.61–2.38)	2.14 (1.74–2.63)

CI – confidence interval, ref – reference, y – years

*Presented as actual sample sizes with weighted percentages.

†Adjusted for all structural and intermediary determinants included in the model.

**Figure 2:**
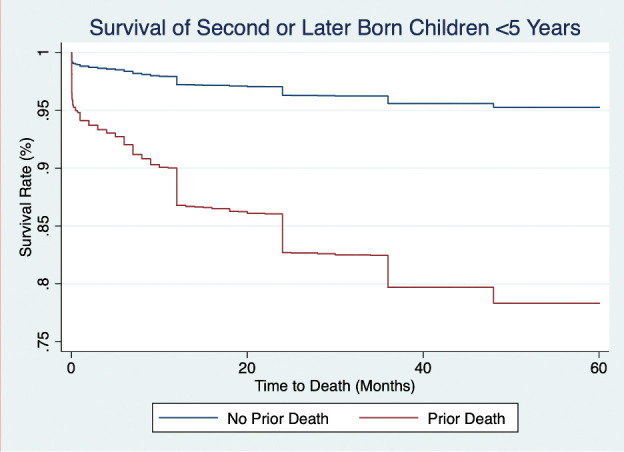
Survival rate for second or later order children in northern Togo in the last 10 years (2008–18) by prior sibling death status.

Approximately 572 (5.2%) of the 11 030-second- or later-order children died before their fifth birthday. Children born to women who identified as Temberma or Peul continued to demonstrate higher hazard of under-five mortality compared to Kabiye children (**Table 3**). Hazards of under-five mortality for children who experienced the prior death of a sibling were also significantly higher than those without a prior sibling death in model 2 (aHR = 5.02; 95% CI = 4.23–5.97) (**Figure 2**).

**Table 3:** Model 2, structural socioeconomic and intermediary biological determinants of under-five mortality for second or later order births in northern Togo in the last 10 y (2008–18)

	Second or later order births in the last 10 y (n = 11 030 (100.00%)), n*	Deaths under-five y (n = 572 (5.15%)), n (%)*	Unadjusted hazard ratio (95% CI)	Adjusted hazard ratio (95% CI)†
**Structural/socioeconomic determinants**				
District				
*Binah*	1740	64 (4.12)	1.00 (ref)	1.00 (ref)
*Bassar*	2501	129 (5.68)	1.36 (1.04–1.78)	1.07 (0.78–1.48)
*Dankpen*	3663	225 (5.85)	1.39 (1.09–1.76)	0.98 (0.67–1.44)
*Keran*	3126	154 (4.56)	1.12 (0.87–1.44)	0.71 (0.47–1.06)
Maternal religion				
*Animist*	7567	412 (5.34)	1.00 (ref)	1.00 (ref)
*Muslim*	732	32 (4.87)	0.94 (0.66–1.33)	0.70 (0.45–1.09)
*Christian*	2294	114 (5.08)	0.95 (0.79–1.14)	1.00 (0.83–1.20)
*No religion*	437	14 (2.64)	0.50 (0.31–0.81)	0.56 (0.35–0.91)
Maternal ethnicity				
*Kabiye*	1723	56 (3.68)	1.00 (ref)	1.00 (ref)
*Konkomba*	3902	237 (5.79)	1.56 (1.21–2.00)	1.13 (0.77–1.66)
*Temberma*	1920	105 (5.06)	1.40 (1.07–1.84)	1.54 (1.00–2.38)
*Lamba*	1442	67 (4.55)	1.27 (0.92–1.75)	1.20 (0.80–1.79)
*Bassar*	467	24 (5.17)	1.39 (0.96–2.02)	1.20 (0.80–1.80)
*Losso*	648	34 (6.44)	1.74 (1.16–2.61)	1.32 (0.84–2.08)
*Peul*	452	24 (6.49)	1.81 (1.18–2.79)	1.99 (1.17–3.36)
*Sola*	203	15 (5.44)	1.51 (0.92–2.49)	1.33 (0.87–2.05)
*Other*	273	10 (4.04)	1.15 (0.62–2.14)	1.07 (0.64–1.78)
Maternal education level completed				
*None*	7453	411 (5.55)	1.00 (ref)	1.00 (ref)
*Primary*	2759	138 (4.57)	0.86 (0.73–1.02)	0.94 (0.79–1.11)
*Secondary or above*	818	23 (3.43)	0.69 (0.44–1.06)	0.81 (0.51–1.30)
Prior death of a child				
*No*	10 137	404 (3.96)	1.00 (ref)	1.00 (ref)
*Yes*	893	168 (18.62)	5.44 (4.65–6.36)	5.02 (4.23–5.97)
**Intermediary biological determinants**				
Maternal age at time of birth				
*≥25 y*	7651	359 (4.81)	1.00 (ref)	1.00 (ref)
*20–24 y*	2735	171 (6.02)	1.22 (1.04–1.42)	1.13 (0.97–1.32)
*≤19 y*	644	42 (5.55)	1.10 (0.84–1.43)	0.91 (0.70–1.17)
Sex of the child				
*Male*	5678	321 (5.59)	1.00 (ref)	1.00 (ref)
*Female*	5352	251 (4.68)	0.83 (0.73–0.95)	0.84 (0.74–0.96)
Birth status				
*Singleton*	10 646	539 (4.98)	1.00 (ref)	1.00 (ref)
*Multiple birth*	384	33 (9.64)	2.16 (1.55–3.02)	2.33 (1.66–3.26)
Birth order				
*2–3*	9652	495 (5.10)	1.00 (ref)	1.00 (ref)
*>3*	1378	77 (5.49)	1.49 (1.23–1.79)	0.79 (0.63–0.99)
Birth interval				
*≥24 mo*	8807	365 (4.22)	1.00 (ref)	1.00 (ref)
*<24 mo*	2126	187 (8.32)	1.84 (1.59–2.12)	1.51 (1.31–1.74)
N/A	97	20 (19.55)	4.77 (3.28–6.96)	3.41 (2.27–5.13)

CI – confidence interval, mo – months, ref – reference, y – years

*Presented as actual sample sizes with weighted percentages.

†Adjusted for all structural and intermediary determinants included in the model.

### Intermediary biological determinants associated with under-five mortality

**Figure 3:**
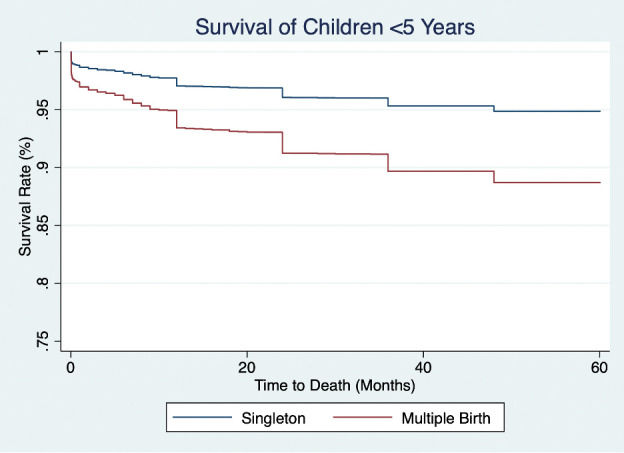
Survival rate for children born in northern Togo in the last 10 years (2008–18) by multiple birth status.

Intermediary biological determinants associated with higher hazards of under-five mortality were maternal age under 25 years; multiple birth status (aHR = 2.27; 95% CI = 1.78–2.90) compared to single births; and higher birth order compared to first born children. Meanwhile, female children had lower hazards of under-five mortality (aHR = 0.80; 95% CI = 0.73–0.89) compared to males (**Figure 3** and **Table 2**).

Maternal age and birth order were not significant predictors of under-five mortality for second- or later-order births in model 2. However, consistent with the analysis of all children, multiples had higher hazards (aHR = 2.33; 95% CI = 1.66–3.26) and female children had lower hazards of under-five mortality among second or later order births. Additionally, children born less than 24 months after a child previously born in the household had higher hazards of under-five mortality than those born 24 or more months later (**Table 3**).

### Intermediary effects of health system utilisation on under-five mortality

Of the 4852 children born in the last two years, 143 (3.0%) died prior to five years of age. In model 3, children whose mothers received any prenatal care compared to no prenatal care (aHR = 0.48; 95% CI = 0.30–0.78) or received high quality compared to inadequate quality prenatal care (aHR = 0.51; 95% CI = 0.29–0.88) had a lower hazard of under-five mortality. There were no statistically significant associations with being delivered in a health facility or receiving any postnatal care (**Table 4**).

**Table 4:** Model 3, health system utilisation and under-five mortality for births in northern Togo the last two years (2016–18)

	Births in the last two years (n = 4852 (100.00%)), n*	Deaths under five years (n = 143 (2.98%)), n (%)*	Unadjusted hazard ratio (n = 4588) (95% CI)	Adjusted hazard ratio (n = 4588) (95% CI)†
Prenatal care				
*No prenatal care*	483	19 (4.51)	1.00 (ref)	1.00 (ref)
*Any prenatal care*	4105	83 (2.08)	0.45 (0.30–0.69)	0.48 (0.30–0.78)
*Missing*	264	41 (11.59)	-	-
Quality of prenatal care				
*Inadequate quality*	3899	94 (2.53)	1.00 (ref)	1.00 (ref)
*High quality*	689	8 (1.16)	0.45 (0.26–0.78)	0.51 (0.29–0.88)
*Missing*	264	41 (11.59)	-	-
Health centre delivery				
*No*	2015	47 (2.46)	1.00 (ref)	1.00 (ref)
*Yes*	2573	55 (2.22)	0.91 (0.67–1.25)	1.17 (0.83–1.64)
*Missing*	264	41 (11.59)	-	-
Postnatal care for baby				
*No postnatal care*	2651	66 (2.64)	1.00 (ref)	1.00 (ref)
*Any postnatal care*	1937	36 (1.93)	0.71 (0.51–0.99)	0.80 (0.55–1.14)
*Missing*	264	41 (11.59)	-	-

CI – confidence interval, ref – reference

*Presented as actual sample sizes with weighted percentages.

†Adjusted for all proxies for health system utilisation included in the model.

## DISCUSSION

We examined potential determinants of under-five mortality in the Kara region of northern Togo between 2008 and 2018 and determined that children whose mothers reported the prior death of a sibling were five times more likely to die before their fifth birthday. Additional structural determinants (such as ethnicity) and intermediary determinants (such as maternal age, sex of the child, multiple birth status, birth order, birth interval, receipt of any prenatal care, and receipt of quality prenatal care) were also important predictors of under-five mortality.

We expanded upon previous analyses in Togo and sub-Saharan Africa to show that prior death of a sibling was an important structural socioeconomic predictor of under-five mortality. Analyses of DHS in Bangladesh between 2004 and 2011 and Nepal between 2001 and 2016 reported that under-five mortality was strongly associated with children whose mothers reported the previous death of a child [21,22]. Although there has been significant clustering of sibling deaths observed in LMICs, this appears to be more common in Western, Central, and Eastern African countries [23]. Approximately 40–50% of female DHS respondents born between 1985 and 2003 in multiple African countries reported the death of a sibling before the age of 25 [23]. Additionally, previous death of a sibling before one years of age was associated with infant mortality in Nigeria and Burkina Faso, according to DHS and Demographic Surveillance System data, respectively [24,25]. Previous death of a sibling may be strongly associated with under-five mortality due to other correlated mortality risks among siblings or socioeconomic determinants within households.

In our analysis for northern Togo, we also determined that ethnicity was another structural socioeconomic predictor of under-five mortality. Children whose mothers identified as Konkomba, Temberma, Lamba, Losso, or Peul were more likely to die before their fifth birthday. There is limited evidence available to understand why under-five mortality may differ by ethnic group; however, a recent multi-country analysis determined that overall inequality in under-five mortality between ethnic groups in Togo decreased between 2013 and 2017 [26].

Our findings support those of the 2013 DHS that intermediary biological determinants such as younger maternal age, child male sex, higher birth order, and birth interval less than two years increased the risk of under-five mortality in Togo [12]. They are also consistent with the findings from 33 sub-Saharan African countries, including Togo, which concluded that lower birth interval, higher birth order, multiple birth status, and lower maternal age increased the risk of under-five mortality [7,8]. Multiple birth status was confirmed as a widely examined determinant of mortality across sub-Saharan Africa and reported as the strongest intermediary predictor of mortality in our study, with more than twice the proportion of deaths occurring among twins and triplets compared to singletons [27]. Meanwhile, our results contradict a recent MICS analysis in Togo that demonstrated increased risk of under-five mortality for female births and decreased risk for second or later order births; however, these findings are not supported elsewhere [6,28].

As illustrated in the WHO Conceptual Framework for Action on the Social Determinants of Health, utilisation of the health system can mitigate the risk associated with intermediary determinants of mortality [18]. In Togo, where access to quality health care is limited due to both a lack of trained health care professionals and an unequal distribution of resources, we expect health care utilisation to play a role in under-five mortality [12]. In this study, the receipt of any prenatal care and quality prenatal care were identified as having protective effects against child death, which aligns with the previously described MICS analysis, despite examining differing measures of utilisation (i.e. attending the four WHO-recommended prenatal visits) [28]. Facility-based delivery and postnatal care were not associated with under-five mortality in this study, which contradicts previous analyses conducted in sub-Saharan Africa [7,8]. However, this is not surprising given that we included baseline data prior to the launch of a targeted primary care programme in a region with historically poor access to care. We expect rates of facility-based birth to increase after exposure to our integrated primary care programme intervention, which may result in reduced child mortality over time. Despite this finding, previous research supports the need for improvements in coverage in these priority maternal and child health areas [29]. Progress towards health system access and utilisation is especially relevant for high-risk pregnancies with other studies demonstrating the need for improved detection of and guidance on care for pregnancies with multiples [30–32].

### Limitations

There are several limitations to our estimation of under-five mortality and analysis of determinants associated with child mortality. Household surveys relied on respondents’ self-report and were completed after the occurrence of reported child births and deaths, which may subject the analysis to recall and social desirability bias. This may have the greatest effects on the reported number of births per household, number of deaths per household, date of birth per child, and age at death per child. There were additional limitations in the analysis of health system utilisation due to limited eligibility criteria (i.e. recent births) and missing values (n = 264), which limited the sample size. This may further explain why facility-based births were not associated with under-five mortality. There was also no updated census or available child mortality registry to compare weighted population estimates and mortality trends determined from our study and validate our findings. Finally, while the measures we investigated were based on the available literature and theory, it is always possible that results are confounded by unmeasured factors.

## CONCLUSIONS

Our study identified several determinants associated with under-five mortality that could be targeted to advance under-five mortality, most notably access to high quality of care, in Togo and similar LMICs. We recommend continued use of data to drive and focus improvement activities by both governmental and non-governmental organisations alike to translate these insights into more equitable progress. Further research is needed to better understand the potential mechanisms driving the relationship between socioeconomic determinants and under-five mortality globally, across sub-Saharan Africa, and nationally in Togo.
